# HSP-Target of Therapeutic Agents in Sepsis Treatment

**DOI:** 10.3390/ijms20174255

**Published:** 2019-08-30

**Authors:** Anderson Vulczak, Carlos Henrique Rocha Catalão, Luiz Alexandre Pedro de Freitas, Maria José Alves Rocha

**Affiliations:** 1 Department of Basic and Oral Biology, School of Dentistry of Ribeirão Preto, University of Sao Paulo, Ribeirão Preto, SP 14040-904, Brazil; 2 Department of Neurosciences and Behavioral Sciences of Ribeirão Preto Medical School, University of Sao Paulo, Ribeirão Preto, SP 14040-900, Brazil; 3 Department of Pharmaceutical Sciences, School of Pharmaceutical Sciences of Ribeirão Preto, University of São Paulo, Ribeirão Preto, SP 14040-903, Brazil

**Keywords:** chaperone, systemic inflammation, immune response, organ dysfunction, heat shock protein

## Abstract

Sepsis is a syndrome characterized by a dysregulated inflammatory response, cellular stress, and organ injury. Sepsis is the main cause of death in intensive care units worldwide, creating need for research and new therapeutic strategies. Heat shock protein (HSP) analyses have recently been developed in the context of sepsis. HSPs have a cytoprotection role in stress conditions, signal to immune cells, and activate the inflammatory response. Hence, HSP analyses have become an important focus in sepsis research, including the investigation of HSPs targeted by therapeutic agents used in sepsis treatment. Many therapeutic agents have been tested, and their HSP modulation showed promising results. Nonetheless, the heterogeneity in experimental designs and the diversity in therapeutic agents used make it difficult to understand their efficacy in sepsis treatment. Therefore, future investigations should include the analysis of parameters related to the early and late immune response in sepsis, HSP localization (intra or extracellular), and time to the onset of treatment after sepsis. They also should consider the differences in experimental sepsis models. In this review, we present the main results of studies on therapeutic agents in targeting HSPs in sepsis treatment. We also discuss limitations and possibilities for future investigations regarding HSP modulators.

## 1. Introduction

Sepsis is a syndrome induced by a dysregulated inflammatory response resulting from an interaction between the host and infectious agents, with consequent organic dysfunction [1,2]. Despite the advances in therapeutic strategies, the high sepsis mortality rate is mainly caused by multiple organ failure and hypotension [3,4,5,6]. Hence, early initiation of treatment is crucial for the preservation of multiple organ functions [1,7], and the success in sepsis treatments is related to improvements in intensive care and, especially so, to early diagnosis based on clear clinical and biological definitions of sepsis [8].

Recent data report short-term mortality of 45% to 50% [9], and half of all survivors may have long-term cognitive decline following sepsis [10]. Its incidence is estimated at 437 cases per 100,000 in the US population, exceeding admissions for acute coronary syndrome or stroke [11]. Due to its great social and economic impact, sepsis appears as a major public health problem. Among all the conditions treated in US hospitals, it corresponds for nearly US $ 24 billion in annual healthcare costs, representing 6.2% of the costs associated with hospitalizations [12].

Although some epidemiological reports show a reduction in sepsis mortality rate [4], the current lack of therapies that directly target the disease suggests that further reduction in mortality is likely related to improvements in the early treatment of sepsis with antibiotics and resuscitation, besides improvements in critical care [7]. Despite investigations on the use of anti-inflammatory, antioxidant, or immune enhancement therapies [1] in the treatment of sepsis, there is no direct correlation between such treatments and robust improvements in sepsis patients.

In recent research efforts directed towards new ways of treating sepsis, analyses on the expression of heat shock proteins (HSPs) with recognized cytoprotective function under conditions of cellular stress [13,14,15] have become a topic of great interest in the development of new sepsis treatments [13,16,17,18,19,20]. HSPs can be activated under conditions of oxidative stress, inflammation, hypoxia, and fever [13,21,22,23], and their role as chaperones is of importance for the functional maintenance of cytosolic proteins [21,22]. Furthermore, HSPs detected in the extracellular space were seen to be involved in immune response signaling [23,24], and their levels were associated with mortality in sepsis [5]. Therefore, the use of therapeutic agents capable of modulating HSP activation in sepsis conditions has been investigated [19,20,25,26,27,28]. Yet, despite promising results, the heterogeneity of the study designs makes it difficult to interpret these data. Furthermore, experimental designs using such therapeutic agents should go beyond metabolism aspects and include questions concerning the development of the immune response in sepsis. It is also important to analyze the role of HSPs with respect to their intra- or extracellular localization. With this in mind, investigations on HSP modulatory agents can generate even more promising results and lead to the development of new strategies for sepsis treatment.

## 2. Sepsis

The word sepsis is a term derived from the Greek verb *sêpsis* that means “putrefaction” or the “decay of organic matter” [3,29]. The presence of pathogenic microorganisms in normally sterile tissues, fluids, or body cavities can lead to infection. Every infectious process triggers an inflammatory response of the host, whose magnitude may differ in each individual [30]. Recently, a conference (The Third International Consensus Definitions for Sepsis and Septic Shock [Sepsis-3]) proposed to update terms, concepts, and parameters used in the identification of steps related to sepsis. Under this consensus, sepsis is now defined as a life-threatening organ dysfunction caused by a dysregulated host response to infection [2]. The main changes proposed by this third consensus conference were the adoption of the SOFA (Sequential Organ Failure Assessment) to diagnose organ dysfunction based on points in a score at least two points consequent to the infection [31,32]. Additionally, the “rapid SOFA score” (qSOFA) was proposed as a screening tool to be used at the bedside to quickly identify, among patients with infection, those with sepsis or those likely to develop it [2]. Due to the lack of prospective validation of the qSOFA, this tool should be used as a predictor of mortality and not as a diagnosis or immediate prognosis of sepsis [32].

In this regard, sepsis is a complex syndrome, with several interconnected and unbalanced organic systems, making both treatment and experimental research models challenging. Several experimental models have been developed to study pathophysiological aspects of sepsis [33,34]. Among these, peritonitis induced by cecal ligation and puncture (CLP) in rodents has become the most widely used model for experimental sepsis [33,34,35,36]. In brief, CLP is done by ligation below the ileocecal valve after midline laparotomy, followed by needle puncture of the cecum [37]. The CLP model is considered as the gold standard because it presents a polymicrobial infection that results in endotoxemia with typical symptoms of sepsis or septic shock, such as hypothermia, tachycardia, and tachypnea, and thus, closely resembles what is observed in patients [37,38]. Although the CLP sepsis model is most similar to clinical sepsis, its morbidity and mortality are unstable due to many factors, such as the extension of cecum ligation, diameter of the needle, and the number of punctures [37,39,40].

In addition to bacterial peritonitis by CLP, models such as intravascular infusion of endotoxin or live bacteria, soft tissue infection, pneumonia or meningitis models have been used to mimic human sepsis [41]. Although the injection of lipopolysaccharide (LPS) can be standardized and is a widely used model of endotoxemic shock, it is useful only for characterizing response patterns and treatments directed against one particular microbe [42]. Models of bacteremia or endotoxemia are of restricted relevance to clinical sepsis, which is commonly polymicrobial, encompassing gram negative and -positive bacteria, as well as aerobic and anaerobic species [43,44,45,46]. Moreover, this model shows a pattern of release of inflammatory and hormonal mediators that differ from the one observed after CLP-induced peritonitis that more closely resembles what is observed in patients [38,47,48,49].

As a multifactorial condition, sepsis reflects the interaction of infectious, immunological, endocrine, hemodynamic, cardiovascular, and even genetic components [50,51,52]. These interactions may lead to an exaggerated response of the organism with the synthesis and action of several inflammatory mediators that produce important physiological alterations [30,53,54]. Not surprisingly, there is controversy about the immune response in sepsis, but it is generally accepted that the immune response in sepsis has an initial hyperinflammatory phase that progresses to a prolonged later immunosuppressive phase [7,55]. This occurs because the cells of the innate immune system release high levels of proinflammatory cytokines, which can even cause the individual’s death at the onset of sepsis due to the hyperinflammatory response [56,57]. In the case of persistent sepsis, it is recognized that failure in both the innate and adaptive immune response leads to immunosuppression, with consequent death due to the development of secondary infections [55]. Sepsis is commonly divided into two sequential phases, in which the first phase is characterized by an initial and reversible hyperinflammatory response, while the second phase is characterized by immunosuppression, usually with organic dysfunction [1,7]. However, evidence has shown that both pro-inflammatory and anti-inflammatory agents are released in the initial phase of sepsis [6,55,58]. Intriguingly, patients who died due to sepsis presented enhanced immunosuppression [59]. In this case, it is assumed that the prolonged activation of the innate immune response would be responsible for organ damage, consequently leading to the death of the individual in the late phase of sepsis [55,58].

Cellular damage and organic dysfunction occur when the immune response is generalized. Although the mechanisms underlying cell injury are not yet fully understood, they are likely related to an oxygen deficit, cell injury by inflammatory mediators, and an altered rate of apoptosis [1,7,45,55,58,60,61]. Regardless, the literature in studies involving sepsis are increasing, treatment options are still rather scarce. Administration of antibiotics, early identification of the source of infection, immediate resuscitation, and multidisciplinary care teams are widely accepted as appropriate care [1,7]. In addition to antimicrobial agents and vasopressors used in the treatment of sepsis, there are investigations using therapeutic agents, such as naloxone, statins and N–acetylcysteine, methyltiouracil [1]. Considering the condition of metabolic and cellular stress caused by sepsis, studies on the role of heat shock proteins [13,15,18,62,63] have also provided valuable data for the understanding of sepsis, as well as generating new perspectives for treatment.

## 3. Heat Shock Proteins and Sepsis 

The exposure of cells to stress conditions, such as hyperthermia, hypoxia, oxidative stress, tissue damage, and infections, require a rapid and efficient response to allow cell survival, and the main proteins expressed in immediate response to such conditions are heat shock proteins (HSPs) [16,64]. Based on molecular weight, HSPs are classified into several families, including HSP110, HSP90, HSP70, HSP60, and small HSPs, such as HSP40 and ubiquitin [21,65,66] (Table 1). HSPs are among the most conserved cellular proteins and function as molecular chaperones. They are located in the cytoplasm and in several organelles, where they act in the stabilization of proteins [18,23], besides also being mediators of the inflammatory response when present in the extracellular environment [15,67]. In this regard, HSPs have been investigated in the context of several inflammatory conditions, such as diabetes [68,69,70], arthritis [71,72], cancer [73,74,75], and sepsis [5,16,76].

In the chaperone function, HSPs regulate the folding, unfolding, solubilization, transport, biosynthesis, and assembly of cellular proteins [22]. Thus, HSPs are of great importance to maintain the conformation of cellular proteins, for intracellular protein homeostasis, and for preserving cellular viability during cellular stress conditions [16,23,65]. Following cellular stress, HSPs are of importance in protein refolding, preventing the aggregation of deformed proteins. Alternatively, they aid in the proteasomal degradation of irreversibly damaged proteins [23,77]. In the absence of cellular stress, heat shock proteins are present in low amounts and play diverse roles in cell maintenance [23,78]. 

Although HSP90 [75,82] and HSP27 [15,83] are associated with cell protection, most of the evidence indicates that members of the HSP70 family are the most important ones in protective role for cells [22,23]. HSP70 is able to interact transiently with peptides during protein synthesis, bringing the new protein into its native, functional conformation [78,84]. In addition, during thermal stress, HSPs can accumulate at the cell membrane, favoring the maintenance of membrane fluidity in response to thermal stress [85] or act as signaling receptors for immune cells [15,74,86]. Moreover, the functions of HSPs are not limited to the intracellular environment because they can be released into the extracellular space where they signal to immune cells [15,24]. 

Therefore, HSPs play a role in the activation of immune cells, and the proposed mechanism is that they can signal to immune cell when tissues are damaged due to infection or inflammation [15,24,71]. HSP70 and HSP90 have been identified as key regulators of the immune response, capable of providing signals to the immune cells even in the absence of immunogenic peptides [15,17,75]. This ability to activate the immune response occurs when HSPs, mainly HSP70 and HSP90, are presented on the cell surface [15,23]. HSPs are expressed on the surface of cells that are infected by a virus or bacteria, on cells of patients with autoimmune disease, or on tumor cells but not on the surface of normal cells [87]. In humans, their presence in serum is associated with stress conditions, including inflammation, bacterial, and viral infections [23].

Although the mechanisms for HSP expression on the cell surface are unclear, they allow recognition by NK cells and cytotoxic T lymphocytes [63,88]. HSP70 was seen to activate macrophages or dendritic cells, besides stimulating cytokine production by monocytes and enhancing the proliferation and cytotoxicity of NK cells [89,90,91]. There is also evidence indicating the presence of a specific HSP70 protein receptor on the surface of macrophages and monocytes [92]. Additionally, the release of cytokines and chemokines by T-Cell and antigen-presenting cell can be to modulated by HSPs as well as the maturation and migration of dendritic cells [93].

However, there is controversy about the impact and role of HSPs in the activation and modulation of both innate and adaptive immunity [16,94,95]. Accumulating findings indicate that HSPs may also attenuate the inflammatory response [96,97,98]. In this regard, intracellular HSPs may have an anti-inflammatory function through inhibition of the pro-inflammatory NF-B pathway [67,70]. Moreover, they are linked with anti-inflammatory responses, by activating regulatory T cell (Tregs) and increasing IL-10 release [71,86,99]. Preclinical and basic studies have described an immunosuppressive activity of some HSPs [86,100,101,102,103]. Among the mechanisms of regulation of the immune response, HSPs were seen to be able to activate the expansion of regulatory T-cells and helper T-cells (Th2), both with anti-inflammatory activity. In addition, the inhibition of T (Th1) cells with pro-inflammatory activity can also be attributed to the action of HSPs [86,101,102,103,104,105]. Therefore, depending on their location, the HSPs may have distinct functions, either a pro-inflammatory or anti-inflammatory response, besides their role as chaperones that preserves the function of other proteins inside the cell.

HSPs, especially the HSP70 family, have been investigated in sepsis conditions, and the results provide evidence for a protective role against organ damage and enhancement in survival in experimental models [14]. This was observed independent on whether the experimental model of sepsis was CLP or LPS injection and despite heterogeneity in the analysis of molecular pathways. What was commonly seen was a role for HSPs in the reduction of proinflammatory cytokines, inhibition of NFκB, reduction in organ damage, and increased survival [17,20,106]. Furthermore, clinical studies indicate a relationship between the patient’s serum oxidative damage with increase HSP70 serum levels, corroborating with a role in the infection and respective mortality [5]. In this regard, the oxidation of blood plasma components, such as hormones, proteins, peptides, and other active substances are rarely considered as factors in sepsis and septic shock [61]. In this sense, treatment with antioxidants or substances linked to the HSP family could represent new ways in the attempt to find strategies for the treatment of sepsis.

## 4. HSPs as Targets of Therapeutic Agents in Sepsis Treatment

Despite advances in the understanding and management of sepsis, there is no specific therapy yet for sepsis in clinical practice, and in this context, the modulation of HSPs and their activity by therapeutic agents may generate new treatment options [17,19,76,107]. 

Studies using glutamine administration, an important amino acid to homeostasis and metabolism immune cells [108], demonstrated positive effects in sepsis. In the CLP experimental model of sepsis, an intravenous administration of glutamine after induction of sepsis increased HSP70 and HSP25 expression, attenuated lung injury, and enhanced survival [109]. After induction of sepsis by LPS, an intravenous administration of glutamine led to an increase in HSP70 expression in lung tissue, as well as in macrophages resident in the lung. In addition, lactate accumulation in lung tissue was similar to the control group, indicating that glutamine attenuated metabolic dysfunction [110]. In another study, Wischemeyer and colleagues observed that the intravenous administration of glutamine increased HSP70 expression in the lung, heart, kidneys, and colon, while HSP25 was increased in the heart, liver, and colon after 6 h of LPS injection in rodents. Furthermore, none of the animals had died at 20 h following LPS injection [111]. The animals treated intraperitoneally with glutamine prior to sepsis induction by LPS also showed increased HSP70 expression, an inhibited translocation of NFκB from cytoplasm to the nucleus, and reduced apoptosis in brain tissue [107]. On the other hand, Cruzat and colleagues demonstrated that animals inoculated with LPS, without treatment, showed an increased expression of HSP27, HSP70, and HSP90 but no change in the mRNA levels of HSPA1, HSPA2, and HSF-1 in gastrocnemius muscle [19]. When the animals were supplemented orally with L-glutamine, however, the basal tissue expression of HSP27, HSP70 and HSP90 proteins was maintained, and for IL-1β and TNFα there was a decrease [19].

In clinical studies, previous infusion of glutamine for 10 h in an endotoxemia model induced by LPS endovenous injection showed no changes in HSP70 in isolated leukocytes [25]. In addition, in vitro research with human cells demonstrated that high doses of glutamine suppressed HSP72 expression, but had no effect on cytokines [17]. Furthermore, glutamine depletion during thermal stress in cells (in vitro) reduced the expression of HSP70 and lymphocyte responsiveness [26]. Nonetheless, even though an association between glutamine and HSPs has been analyzed and shown in different tissues and serum, the molecular mechanisms underlying the action of glutamine on HSP expression, either in the experimental animal models, humans, or cell cultures are still unclear. 

Other therapeutic agents have also been used in the investigation of HSP and sepsis. After sepsis model with CLP, the intravenous injection of sodium arsenite, an inorganic salt with properties of an antibacterial agent [112], increased the expression of HSP72 in the lungs and increased survival by 84% after 24 h in rodents [27]. Dehydroepiandrosterone (DHEA), a naturally occurring steroid that has been shown to protect mice from bacterial and viral infections, was also investigated in sepsis. DHEA has immunomodulatory properties and when administered subcutaneously post sepsis resulted in increased HSP70 expression in the lung and spleen of animals with sepsis, followed by an attenuation in the release of TNF-α in plasma, and a reduction in mortality [113]. Celastrol is a chemical compound with antioxidant and anti-inflammatory properties isolated from the root of *Tripterygium wilfordii* [114]. When administered intravenously, before LPS-mediated induction of sepsis, it increased the expression of HSP70 and of the transcription factor HSF-1 in heart and the aorta, suppressing oxidative stress and inflammatory responses, identified by the attenuation in iNOS and NFκB [115]. Intraperitoneal pretreatment with zinc, an essential trace element for the maintenance of immune function [116], increased HSP70 mRNA levels and reduced apoptosis in splenocytes of septic animals [28]. Moreover, the zinc treatment did not change IL-6, IL-1β and TNFα, but decreased IFN-γ levels in serum. Interestingly, in splenocytes the production of IFN in the treated group was higher than in the LPS group [28]. Interestingly, oral pre and post treatment with curcumin, which is derived from the tropical plant *Curcuma longa* L. (Zingiberaceae) and has anti-inflammatory actions [117], was also seen to reduce the serum expression of HSP 70 and IL-6, as well as IL-1β proinflammatory cytokines despite to show an increase in serum NO following 24hs of sepsis induced by CLP [20]. Therefore, the use of a variety of therapeutic agents associated with HSP in sepsis has been investigated, but the interpretation of the results is still challenging (Table 2).

## 5. Current Status of Knowledge on HSP Modulation by Therapeutic Agents in Sepsis

Despite the use of a variety of therapeutic agents, the mechanisms of action of potential modulators of HSP activity that demonstrated beneficial effects in reducing damage in sepsis are still unclear. One of the difficulties is the heterogeneity in experimental designs in the studies on the impact HSP activity modulating therapeutic agents in sepsis. Although the functions of HSPs have been described both in the intracellular and extracellular environment, no study that proposed to use substances for the treatment of sepsis has performed both intra- and extracellular HSP analyses. Only the study by Silva and colleagues [20] analyzed serum HSP levels, while most of the other studies focused on HSP expression or activity in lung tissue [27,109,110,111,113]. Nevertheless, it is equally important to consider the role of HSPs in the extracellular environment in the modulation of the immune response [24,62,70] because the crosstalk between intra- and extracellular parameters may proof valuable for the understanding of the role of HSPs and of therapeutic agents targeting these in the treatment of sepsis.

Sepsis is characterized by imbalance between pro-inflammatory and anti-inflammatory responses [118], with an amplification of the initial host response to infection and subsequent deregulation [57]. In this respect, studies that aimed at linking compounds to enhanced serum, plasma, or tissue HSP concentrations analyzed parameters of the immune response that focused only on the quantification of a few pro-inflammatory cytokines, demonstrating that the treatment was able to decrease serum TNFα, IL-1β and IL-6 [19,20,113,115], as well as IFN-γ [28]. However, in septic animals treated with zinc, no differences in serum TNFα, IL-1β, and IL-6 levels were observed when compared with control animals [28]. Other study analyzed the time course of serum or plasma cytokines during sepsis development [20]. Both studies observed that after 24 h, but not at 6 h post induction, proinflammatory cytokines were reduced. Herein, the inflammatory response triggering and cytokine-mediated signaling pathways included the activation of important transcriptional factors for the immune response, including NFκB [15,118], which has been shown to be attenuated by treatment with glutamine [107] and celastrol [115].

Although sepsis is closely linked to the inflammatory response, the number of studies that analyzed immune response parameters in sepsis treatment with compounds that potentially enhance the role of HSPs is scarce. Moreover, these studies primarily analyzed pro-inflammatory markers [19,20,28,107,113,115], while the acute inflammatory response depends on both pro-inflammatory and anti-inflammatory cytokines [55,118,119]. Thus, it is important to investigate the role of cytokine networks in the immune response in both septic tissue and serum or plasma, since cytokines may increase or suppress the production of other cytokines [119]. However, inflammatory markers such as TNFα, IL-1, or IL-10 may exhibit variation and inconsistencies in gene expression, which may be a consequence of the heterogeneity of the individuals’ immune response [118].

Depending on the developmental phase of sepsis, parameters of the immune response also show important differences [55] that need to be considered. In the acute phase, the cytokines IL-6, IL-8, MCP-1, and IL-10 appear to play a key role in the patient’s prognosis [119]. Interestingly, the initial phase of hyperinflammation may be followed or overlapped by a prolonged state of immunosuppression [55,118], reported as sepsis-induced immunoparalysis [120]. Consequently, this compromises innate and adaptive immune responses and has an important role in pathogenesis, including damage reduction in survivors [55,76]. In addition, one study demonstrated a reduction in inflammatory markers at 24 h after the experimental induction of sepsis [20]. Therefore, the host response in sepsis is complex, with an interaction of pro-inflammatory and anti-inflammatory factors during sepsis development. Hence, the investigations need a robust experimental design to improve the understanding of sepsis and to reveal new treatment possibilities.

## 6. Limits of Current Research Concerning Potential HSP Modulators in Sepsis

Investigations on therapeutic agents capable of modulating HSPs in sepsis complications present certain limitations. Intriguingly, a number of studies reported that the administration of several therapeutic agents during sepsis complications resulted in the modulation of HSP expression in serum [20,113] and target organs [19,27,121]. However, it is difficult to establish how the HSP expression is induced by therapeutic agents, since their expression appears to be sensitive to many agents, including glutamine [19,25,26,107,111], curcumin [20], celastrol [115], zinc [28], DHEA [113], and sodium arsenite [27].

For instance, two studies that analyzed glutamine as an HSPs modulator used quercetin [109,110], an HSPs inhibitor, making the results more robust when compared with another study that did not use an HSP inhibitor. Even though quercetin has no influence in HSF-1 DNA-binding [122], its use may contribute to the understanding of the actions of HSP in tissues and serum. Moreover, despite genetic compensation mechanisms [123], the development of experimental designs with knockout models may be valuable for the identification of metabolic pathways that are able of regulating HSP actions in sepsis. The administration of therapeutic agents may also impact on signaling pathways that influence several cellular processes, including growth, differentiation, stress response and adaptation, as well as hormonal and immunological responses [124,125]. Therefore, by the identification of genes that are up- or down-regulated, molecular interactions and metabolic pathway can be analyzed and contribute to a better understanding of the role of HSPs in sepsis.

Heterogeneity in experimental design is an important limiting factor for the comparison and analysis of the effect of therapeutic agents used in the treatment of sepsis. Moreover, the HSP localization can have different effects, for example, intracellular HSPs have cytoprotective effects, while extracellular HSPs can activate the immune system [24]. However, studies on HSP protein expression that discriminate between intra- and extracellular effects are rare, making it difficult to draw firm conclusions, and it is necessary to define in advance whether therapeutic agents used for the treatment of sepsis will target intra- or extracellular HSPs.

In addition, the administration of therapeutic agents in the treatment of sepsis should consider aspects of pharmacokinetics and pharmacodynamics, as their effects are related to their concentration, and understanding their action over time can be used to optimize therapy [126,127]. Generally, studies analyzing the administration of HSP modulating agents in sepsis treatment do not describe pharmacokinetic and pharmacodynamic parameters. The main factors that require better interpretation concern dosage and routes of administration [17,25,110,111,128], as well as treatment effects before [20,28,107,113] and after sepsis [19,27,109,110,111,115]. Aspects of absorption, metabolization, distribution, and excretion of therapeutic agents administered during sepsis, as well as their concentration also need to be studied with respect to the different stages of sepsis.

During the acute and late phases of sepsis, alterations in metabolic hormone regulation and in the immune system should be analyzed separately [129]. Additionally, it is generally accepted that the activation of both pro- and anti-inflammatory factors occurs after the onset of sepsis [55,130]. Exacerbated activation of the innate immune response in the initial phase or persistent immunosuppression over time may lead to death. Equally, the persistent activation of innate immunity can also lead to death due to inflammation and organ damage [55]. Current guidelines recommend starting antibiotic therapy within one hour of identification of septic shock, and every hour delay is associated with a 6% rise in mortality [61]. Hence in experimental designs, it is fundamental to relate the treatment parameters over time in association with the immune response.

Studies with humans necessarily present difficulties in sample collection and, especially so, in the control of conditions; therefore, experimental sepsis models are widely used in basic research. As detailed above, animal studies widely employ CLP or an exogenous injection of LPS as experimental models. In the LPS model, the serum cytokine response is transient and reaches a magnitude that is higher than the clinically observed one [131]. In addition, certain treatments reported as effective in the LPS animal model of sepsis failed in clinical trials [132,133,134]. Although the LPS injection model has contributed to reveal and explain the activation pathways involved in the pathogenesis of sepsis, it does not represent the typical episodes of clinical sepsis but likely is an appropriate model of endotoxemic shock [41,132,134]. Thus, the challenge with LPS is not an appropriate model for replicating human sepsis [135]. The CLP model is also widely used in sepsis research, mainly because it approximates clinical observations in inflammatory conditions induced by polymicrobial peritonitis, perforated appendicitis, diverticulitis, bacteremia, and systemic sepsis [41,47,132,133,136,137,138]. In the CLP sepsis model, the inflammatory focus, polymicrobial infection, and pattern of release of inflammatory mediators is more complex than in the LPS model of sepsis [47,131,139,140]. Furthermore, sepsis lethality can be controlled, either by the LPS dosage, and in CLP by the size of the needle and by the number of punctures [40,141]. Thus, the analysis of the efficacy of therapeutic agents in modulating HSP expression or activity or other potential pathways should consider the magnitude of induction of sepsis.

In consideration of these difficulties, despite many clinical trials, no approved drug or therapeutic agent is yet available for use in sepsis [142]. The different HSP functions and molecular effects that their modulation may cause, combined with the complex biology of sepsis, are still a challenge for sepsis treatment.

## 7. Future Perspective on HSP Modulators in Sepsis Treatment

As a therapeutic targets, HSPs have aroused great interest, and investigations on their role in sepsis are ongoing. Because sepsis causes immune and metabolic alterations, oxidative stress, accumulation of damaged proteins, which all lead to organ failure and deregulated inflammation, cytoprotective machinery involving HSPs as well as their signaling function, is being investigated for use in strategies of sepsis treatment. Experimental studies have shown a protective effect for some HSP-modulating compounds during sepsis. Among these, glutamine is the most investigated one, and data demonstrate that it is effective in sepsis. Other studies have focused on the analysis of HSP70 and other members of the HSP family and their essential role in cytoprotection [14,23], and activation of the immune response [23,89]. In this respect, other heat shock proteins, such as HSP25 [109,111], HSP27, and HSP90 [19] also appear to be sensitive to therapeutic agents and may become of interest in future investigations. The heterogeneity of experimental models and procedures still represents a challenge to data interpretation. Nevertheless, a minimum quality threshold in animal model sepsis studies was proposed by experts in an international consensus, which proposed guideline points as “best practices” to be implemented [135]. Hence, regardless of which therapeutic agent will be analyzed, future research needs to consider the appropriateness and respective limits in experimental models and design for sepsis induction, the effective dosage for sepsis treatment, the appropriate route of administration, the impact on the immune response. Furthermore, consideration must be given concerning the effect of the respective therapeutic agent on HSP modulation in both the intracellular and extracellular environment and according to the stage of sepsis development. Nevertheless, despite these difficulties, studies on HSP modulators may represent the next advance in sepsis-related research.

## Figures and Tables

**Table 1:** Characteristics of Heat Shock Proteins usually investigated in sepsis.

Family	Heat Shock Protein (Molecular Weight)	Localization	Function
Small HSPs [79,80]	HSP 25 * (22 kDa)	Cytosol-nucleus [80]	Chaperone [79] Immune cell activation [15]
	HSP 27 (22 kDa)	Cytosol-nucleus [79,81]	
	HSP 40 (38 kDa)	Cytosol [79,81] nucleus [81]	
HSP 60 [79]	HSP 60 (61 kDa)	Cytosol-mitochondria [79,81]	Chaperone [79] Immune cell activation [15,16]
HSP 70 [79]	HSP 70 (70 kDa)	Cytoplasm [79,81]-nucleus [81]	Chaperone [79] Immune cell activation [15,16]
	HSP 72 (71 kDa)	Cytosol-Nucleus [81]	
	HSP 73 / HSC 70 (71 kDa)	Cytosol [79,81] Nucleus [81]	
HSP 90 [79]	HSP 90A (86 kDa)	Cytosol [79,81] Nucleus [81]	Chaperone [79] Immune cell activation [15,16]
	HSP 90B (84 kDa)	Cytosol [79,81] Nucleus [81]	
	GRP94 (92 kDa)	ER [79,81] Cytosol [79]	
Large HSPs [79]	HSP 110 (96 kDa)	Nucleus [81] Cytosol [79]	Chaperone [79]

* HSP25 in animals is called HSP27 in human cells.

**Table 2:** Therapeutic agents used in experimental models of sepsis as HSP modulators.

Therapeutic Agent	Protocol (Pre/Post-Sepsis)	Dosage	Sepsis Model	HSP Expression
Glutamine [109]	1 h (post)	[400 mg/Kg] i.v.	CLP	↑
Glutamine [110]	5 min (post)	[750 mg/Kg] i.v.	LPS	↑
Glutamine [111]	10–20 min (post)	[750 mg/Kg] i.v.	LPS	↑
Glutamine [107]	7 days (pre)	[1.346 mg/Kg] i.p.	LPS	↑
L-Glutamine [19]	2 h, 24 h and 45 h (post)	[1000 mg/Kg] oral	LPS	−−
Sodium Arsenite [27]	8 h (post)	[6 mg/Kg] i.v.	CLP	↑
DHEA [113]	6 h (post)	[20 mg/Kg] s.c.	CLP	↑
Celastrol [115]	30 min (pre)	[1 mg/Kg] i.v.	LPS	↑
Zinc [28]	5 days (pre)	[3 mg/Kg] i.p.	LPS	↑
Curcumin [20]	7 days (pre)/2 h (post)	[100 mg/Kg] oral	CLP	↑

CLP: cecal ligation and puncture; LPS: injection of lipopolysaccharide; i.v.: intravenous; i.p.: intraperitoneal; s.c.: subcutaneous; ↑ increased expression; −− no change.
